# Detection of Bhanja Bandavirus in Patients with Neuroinvasive Disease of Unknown Etiology in Croatia

**DOI:** 10.3390/microorganisms11092155

**Published:** 2023-08-25

**Authors:** Tatjana Vilibic-Cavlek, Vladimir Stevanovic, Stjepan Krcmar, Vladimir Savic, Snjezana Kovac, Maja Bogdanic, Maja Mauric Maljkovic, Dario Sabadi, Marija Santini, Tanja Potocnik-Hunjadi, Mahmoud Al-Mufleh, Ljubo Barbic

**Affiliations:** 1 Department of Virology, Croatian Institute of Public Health, 10000 Zagreb, Croatia; maja.bogdanic@hzjz.hr; 2 School of Medicine, University of Zagreb, 10000 Zagreb, Croatia; marijasantini.ms@gmail.com; 3 Department of Microbiology and Infectious Diseases with Clinic, Faculty of Veterinary Medicine, University of Zagreb, 10000 Zagreb, Croatia; skovac@vef.unizg.hr (S.K.); ljubo.barbic@vef.unizg.hr (L.B.); 4 Department of Biology, Josip Juraj Strossmayer University of Osijek, 31000 Osijek, Croatia; stjepan@biologija.unios.hr; 5 Poultry Center, Croatian Veterinary Institute, 10000 Zagreb, Croatia; v_savic@veinst.hr; 6 Department for Animal Breeding and Livestock Production, Faculty of Veterinary Medicine, University of Zagreb, 10000 Zagreb, Croatia; maja.mauric@vef.unizg.hr; 7 Department of Infectious Diseases, Clinical Hospital Center Osijek, 31000 Osijek, Croatia; dariocroatia@gmail.com; 8 School of Medicine, Josip Juraj Strossmayer University of Osijek, 31000 Osijek, Croatia; 9 Department for the Immunocompromised Patients, University Hospital for Infectious Diseases “Dr. Fran Mihaljevic”, 10000 Zagreb, Croatia; 10 Department of Infectious Diseases, General Hospital Varazdin, 42000 Varazdin, Croatia; tanja.potocnik.h@gmail.com; 11 Department of Infectious Diseases, County Hospital Cakovec, 40000 Cakovec, Croatia; mahmoud.almufleh@gmail.com

**Keywords:** Bhanja bandavirus, neuroinvasive disease, seroprevalence, Croatia

## Abstract

Background: Although the Bhanja bandavirus (BHAV) is widely distributed in some European countries, human infections are rarely reported. This study analyzed the prevalence of BHAV antibodies in patients with neuroinvasive diseases of unsolved etiology. Methods: A total of 254 Croatian patients who developed neurological symptoms during the four consecutive arbovirus transmission seasons (April 2017–October 2021) were tested. Cerebrospinal fluid (CSF) and urine samples were tested using RT-qPCR. In addition, CSF and serum samples were tested using a virus neutralization test. Results: BHAV RNA was not detected in any samples, while neutralizing (NT) antibodies were detected in serum samples of 53/20.8% of patients (95% CI = 16.0–26.3). In two patients, BHAV NT antibodies were detected in the CSF, indicating a recent infection. Both patients were inhabitants of rural areas in continental Croatia, and one reported a tick bite two weeks before symptoms onset. The seropositivity was high in all age groups (15.2–29.1%). The majority of seropositive patients (94.3%) resided at altitudes less than 200 m above sea level. The prevalence rates correlated positively with population density and negatively with certain climate parameters (temperature, number of hot/warm days). Conclusions: The presented results indicate that BHAV is distributed in Croatia. Further studies are needed to determine the clinical significance of this neglected arbovirus.

## 1. Introduction

Bhanja bandavirus (Bandavirus bhanjanagarense according to 2022 ICTV Taxonomy Release; BHAV) is a tick-borne bunyavirus that belongs to the family *Phenuiviridae*, genus *Bandavirus* [1]. The virus was first isolated in 1954 from the *Haemaphysalis intermedia* tick collected from a paralyzed goat in Bhanjanagar, India [2], while the first human case of BHAV infection was reported in 1974 [3]. A laboratory worker developed a mild febrile disease characterized by slight fever, muscle and joint pain, headache, and photophobia which lasted 48 h. The virus was isolated from blood and a diagnostic increase in neutralizing (NT) antibody titers was demonstrated in a convalescent serum sample [4].

Although the BHAV is widely distributed in central Europe, around the Mediterranean basin, across the Middle Eastern countries to India, and in Sub-Saharan Africa, human clinical cases are rarely recorded [5]. Sheep, goats, cattle, African hedgehogs (*Atelerix albiventris*), and African ground squirrels (*Xerus erythropus*) are the vertebrate hosts of BHAV. Adult animals usually do not show clinical signs; however, the virus is pathogenic for young ruminants causing fever and signs of central nervous system (CNS) involvement. Vectors of the BHAV include metastriate ixodid ticks of the genera *Haemaphysalis*, *Dermacentor*, *Hyalomma*, *Rhipicephalus*, *Boophilus*, and *Amblyomma. Haemaphysalis* ticks are the main vector species in Europe [3]. Eight species of ticks from the genus of *Haemaphysalis* were recorded in Europe and North Africa [6], while six species of these eight were recorded in Croatian fauna [7].

In Croatia, BHAV was isolated in 1974 from *Hemaphysalis punctata* female ticks collected from sheep on Brač Island [8]. It is a three-host tick with a natural life cycle of one to three years. During its immature stages, its hosts are small mammals, birds, and lizards. Adults mainly feed on wild and domestic ungulates and rarely on humans. *Haemaphysalis punctata* is adaptable to different climatic conditions and inhabits various habitats, ranging from cold to mild and humid to dry [9].

In 1974, there was also a report of the first human laboratory BHAV infection in Croatia, followed by two additional laboratory infections recorded in 1977 [10]. After BHAV was retrospectively diagnosed in a patient who presented with meningoencephalitis and spastic quadriparesis in Zagreb, its role as a zoonotic virus was established in 1975. The patient developed symptoms in December and three weeks before the disease onset she had stayed in northern Croatia, a region where *H. punctata* ticks were also found [11].

Seroepidemiological investigations conducted after the virus isolation in Croatia found BHAV hemagglutination-inhibiting (HI) antibodies in 31.5% of the inhabitants of Brač Island (1975 and 1977) [12]. In the other study (1975), BHAV-neutralizing (NT) antibodies were detected in 35.8% (11.6% to 61.3%) of the population from eight villages of Brač Island [13]. Furthermore, HI antibodies were found in 2.2% of residents of the islands around Zadar (Middle Dalmatia) and 1% of the residents of Hvar Island, as well as in 7.1% of the inhabitants of Northern Croatia [14].

However, more recent data on the BHAV prevalence in Croatia are missing. The aim of this study was to analyze the prevalence of BHAV antibodies in patients with neuroinvasive diseases of unsolved etiology who developed the symptoms during the arbovirus transmission season.

## 2. Materials and Methods

### 2.1. Patients

This study included 254 patients with unsolved neuroinvasive infections (‘febrile headache’, meningitis, encephalitis, myelitis) from continental and coastal Croatian regions who developed symptoms during the four consecutive arbovirus transmission seasons (April 2017–October 2021). The median patient’s age was 51 (IQR = 31–67) years. Cerebrospinal fluid (CSF) and serum samples were collected in all patients, while urine samples were available for 98 patients. Sampling was performed during the acute phase of the disease. Viral etiology of the neuroinvasive disease was suspected based on the CSF analysis (pleocytosis, mononuclear predominance, elevated protein level, and normal glucose level).

### 2.2. Methods

CSF and urine samples were tested for the presence of neuroinvasive arboviruses: tick-borne encephalitis virus (TBEV), West Nile virus (WNV), Usutu virus (USUV), Toscana virus (TOSV), Tahyna orthobunyavirus (TAHV), and BHAV. Viral RNA was detected using a real-time quantitative reverse transcription polymerase chain reaction (RT-qPCR): TBEV (Schwaiger and Casinotti, 2003) [15], WNV (Tang et al., 2006) [16], USUV (Nikolay et al., 2015) [17], TOSV (Weidmann et al., 2008) [18], and TAHV (Li et al., 2015) [19].

For BHAV RNA (RT-qPCR), a Brilliant III Ultra-Fast QPCR Master Mix (Agilent Technologies) and Rotor-Gene Q real-time PCR cycler (Hilden, Germany) were used. Viral RNA was extracted using a High Pure Viral Nucleic Acid Kit (Roche Applied Science). The primers targeting the 166 nt region of the C segment of the BHAV genome and probe used were the following: forward primer—GAT GGT TGC ATA CAC TGA, reverse primer—CTT GGC ATC ATC TTT CCA, and probe—FAM-ATC CTT AAG GAG TTC GGT GAG GA-BHQ-1. Reactions were performed in 20 µL reaction volume containing 5 µL RNA, 0.4 µM of each primer, 0.2 µM of probe, 10 µL of 2x Brilliant III Ultra-Fast QRT-PCR Master Mix, and 1 µL of RT/RNase block. The conditions were as follows: 10 min 50 °C, 3 min 95 °C, and 40 cycles of 10 s 95 °C, 10 s 54 °C, and 30 s 72 °C.

Serum and CSF samples were tested for the presence of arboviral antibodies: TBEV/WNV/USUV (ELISA; Euroimmun, Lübeck, Germany), TOSV (IFA; Phlebovirus mosaic, Euroimmun, Lübeck, Germany), TAHV, and BHAV (virus neutralization test; VNT) [20].

BHAV strain IG690 grown in Vero E6 cells was used as an antigen for VNT. Prior to VNT, the antigen was titrated in Vero E6 cells. The virus titer (TCID_50;_ tissue culture infectious dose 50) was calculated using the Reed and Muench formula [21]. Serum samples were heat-inactivated at 56 °C for 30 min. Starting from a dilution of 1:5, serial two-fold dilutions were prepared in microtiter plates. An equal amount (25 µL) of inactivated serum dilutions and 100 TCID_50_ of BHAV were added and the mixtures were incubated for one hour at 37 °C with CO_2_. Finally, 50 µL of 2 × 10^5^ Vero E6 cells/mL were added to each well. The plates were incubated at 37 °C with CO_2_ and, starting from the third day, the inoculated cells were inspected daily for the cytopathic effect. NT antibody titer was defined as the reciprocal value of the highest serum dilution that showed at least 50% neutralization. Virus back titration, negative, and low positive control were included in each run. An NT antibody titer of ≥10 was considered positive.

### 2.3. Statistical Anaylsis

The differences in seropositivity rates according to patients’ demographic/clinical characteristics (gender, age, area of residence, and clinical presentation) were compared using a Chi-square test. The strength of the association between dependent (VNT positivity) and independent variables was assessed by logistic regression. The associations between prevalence rates and climate/meteorological data (mean temperature, altitude, population density, insolation duration, precipitation amount, number of cold, warm, and hot days) were analyzed using linear regression (lm function) in R [22]. Climate data were obtained from the Croatian Meteorological and Hydrological Service [23]. The population density data are available at the official site of the Croatian Bureau of Statistics. *p* value < 0.05 was considered statistically significant. Statistical analysis was performed using Stata version 16 software (StataCorp LLC, Lakeway Drive College Station, TX, USA).

## 3. Results

### 3.1. Prevalence of BHAV Antibodies in Patients with Neuroinvasive Disease

BHAV RNA was not detected using RT-qPCR in any of the tested samples, while BHAV NT antibodies were detected in serum samples of 53 (20.8%) patients (95% CI = 16.0–26.3) with titers ranging from 10 to 40. In addition, antibodies were detected in the CSF of two patients, indicating a recent BHAV infection (in one patient, the serum sample tested negative).

Demographic and clinical characteristics and laboratory results of patients with neuroinvasive disease are presented in Table 1 and Table 2.

The prevalence of BHAV NT antibodies according to demographic and clinical characteristics and risk analysis are presented in Table 3 and Table 4. There was no significant difference in the seropositivity between males (20.6%) and females (22.4%). Analyzing the seroprevalence in different age groups, younger patients were more often seropositive (participants younger than 20 years 29.1%, 20–29-year-olds 25.8%) compared to other age groups (15.2–21.7%). However, these differences were not significant. In addition, no significant differences were observed in individuals residing in urban (19.3%) and suburban/rural areas (26.0%). Interestingly, there were significant differences (*p* < 0.001) according to clinical presentations with significantly higher prevalence rates in patients with meningitis (44.7%) and febrile headache (22.2%) than with meningoencephalitis and myelitis (10.0% each).

### 3.2. Geographic Distribution of BHAV-Seropositive Patients

**Figure 1:**
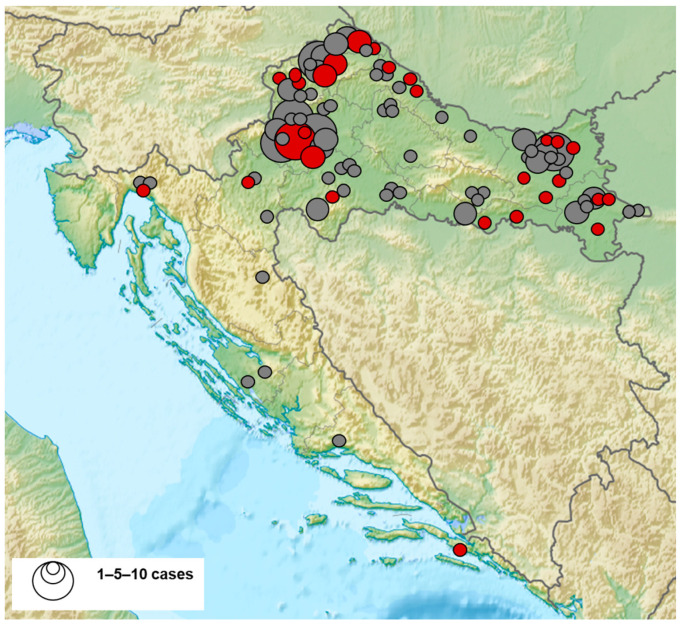
Geographic distribution of Bhanja bandavirus seropositive patients (seropositive patients—red dots, seronegative patients—gray dots).

The geographic distribution of BHAV-seropositive patients is presented in Figure 1. Cases were recorded in 11/14 continental and 2/7 coastal counties. The prevalence of positive patients was higher in the northwestern (39/169; 23.1%, 95% CI = 16.9–30.2) compared to the eastern regions (12/73; 16.4%, 95% CI = 8.8–26) and coastal regions (2/12; 16.6%, 95% CI = 2.1–48.4).

### 3.3. BHAV Prevalence Rates According to Region, Climate and Geographic Data

**Figure 2:**
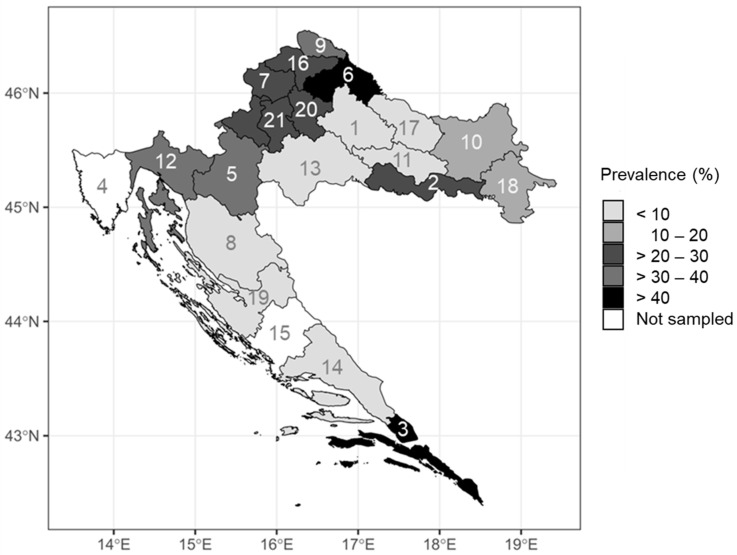
Bhanja bandavirus prevalence rates according to region (numbers in the map represent county labels).

BHAV prevalence rates differed among counties (Figure 2). The geographic and climate data of the counties with detected seropositive patients are presented in Table 5. There was a significant positive correlation between the BHAV NT antibody prevalence rates and population density (*p* < 0.001). In counties with higher population density prevalence rate was higher. Data from the City of Zagreb were removed from the statistical analysis since the population density in this region is a few folds greater than in any other region, making it an outlier.

The vast majority of the BHAV NT antibody-positive samples (94.3%) were collected from patients living at lower altitudes. Only three positive samples were collected from patients living at an altitude higher than 200 m. On the other hand, a significant increase in the prevalence rate with higher altitudes in patients residing under 250 m a.s.l. was observed (*p* = 0.012). In patients residing at altitudes lower than 250 m, there was also a significant negative association between the prevalence rate and mean yearly temperature (*p* = 0.037), the average number of warm days (days with daily maximum ≥25 °C; *p* = 0.010), and hot days (days with daily maximum ≥30 °C; *p* = 0.040). Total precipitation amount (*p* = 0.401), number of cold days (days with daily maximum ≤0 °C; *p* = 0.050), or cumulative insolation duration (*p* = 0.254) showed no influence on prevalence rates (Table 5).

## 4. Discussion

The BHAV presence was confirmed in Croatia in the 1970s and 1980s. However, there are no data on the virus circulation thereafter. In addition, the seroprevalence was analyzed only in the general population, but not in the patients with CNS infection.

In the present study, a recent BHAV infection (confirmed by the detection of NT antibodies in the CSF) was recorded in two patients. In one patient, antibodies were detected in the CSF, while the serum sample was negative. In some patients with arboviral neuroinvasive disease, it was observed that IgM in CSF could appear even earlier than in the serum [24], which could explain the negative result in the serum sample of this patient. Both patients were residents of rural areas, and one reported a tick bite two weeks before symptoms onset. The onset of the disease (July and November) corresponds to the seasonal bimodal activity of adult *H. punctata* ticks. The adults have a bimodal activity with peaks from March to June and September to November [9]. Although, it was observed that an important impact on the interpretation of seasonal dynamics was the methodology used for tick collecting [25].

In addition, BHAV NT antibodies were detected in the serum samples of 20.8% of patients with neuroinvasive disease suggesting the BHAV circulation in Croatia. Since the control group of asymptomatic individuals was not tested it was not possible to compare the seroprevalence in patients with CNS infection with the seroprevalence in the general population, which is a limitation of this study that should be addressed. However, previous Croatian studies showed lower seroprevalence rates in the general population of north Croatia (5.53%), suggesting the potential role of BHAV in the etiology of neuroinvasive infections.

Only a few seroprevalence surveys on BHAV were conducted in Croatia. One study conducted on Brač Island (N 43°18′17″, E 16°39′11″) in 1975 showed a very high overall BHAV seropositivity rate of 31.54%. Seropositive individuals were detected in 8/19 places tested [12]. In contrast, seroprevalence on the neighboring Hvar Island was very low (1%) [14]. Some possible predictors of BHAV foci that could affect the probability of BHAV occurrence may include a) the plant community *Ostryo-Carpinion adriaticum*, which may be found in the elevated center of Brač, does not appear on Hvar; b) *H. punctata* ticks have much lower population densities on Hvar than on Brač; and c) very few domestic ruminants graze on Hvar (typical extensive pastures are entirely absent), in contrast to their large numbers on Brač [3]. A subsequent study (1979–1980) tested the inhabitants of two different ecological areas: residents of North Croatia along the Hungarian border and BHAV seronegative newcomers to that region, as well as newcomers to the areas along the Italian border (Slovenia) who were staying there for a year. The first region has a mild climate and a plain landscape (95–128 m a.s.l.). The second region has a Mediterranean climate and rises to 600 m a.s.l. The mean age of participants in these groups was 18.19, 20.5, and 20.5 years, respectively. BHAV HI antibodies were found in 5.53% of examined natives of North Croatia, while in the newcomers after one year, HI antibodies were confirmed in 3.27% of the samples examined. In addition, HI antibodies were found in 1.95% of samples of youngsters along the Italian border. These results suggested a recent circulation of BHAV in the regions studied. Analyzing the seropositivity according to geographic area, the highest frequency of antibody detection was in the most western parts (7.6%), with decreasing trends toward the east (1.0–2.4%). In addition, the frequency of seroconversion was higher in the western (6.62%) and lower in the eastern regions (0.82 and 1.85%). However, these geographical differences in seropositivity were not statistically significant [25]. The majority of seropositive patients in the present study were inhabitants of continental Croatian regions. Similar to previous studies, the seropositivity was higher in the northwestern than in the eastern and coastal regions (23.1%, 16.4%, and 16.6%, respectively).

Few studies analyze the BHAV seroprevalence in the general population of Europe, mainly in endemic regions, whereas data on the seroprevalence in patients with neuroinvasive disease are very scarce. In a study from Italy (1967–1968), the prevalence of HI antibodies in humans was 1.8% with the majority of seropositive individuals detected in south and central regions [26]. One study from the Czech Republic tested adult patients from four districts presented with meningoencephalitis and “virosis” from 1975 to 1983. BHAV NT antibodies were found in 2.9% of patients [27]. A study published in 1982 analyzed the prevalence of BHAV antibodies in Czechoslovak citizens from two districts in South Moravia (Znojmo and Breclav) and one district in East Slovakia (Roznava). BHAV NT antibodies were detected in 5.4% of participants [28].

The present study showed no significant differences in the BHAV NT antibody prevalence rates between age groups (15.2–29.1%). However, it was interesting that the highest seropositivity rates were observed in the youngest participants (29.1% in individuals younger than 20 years and 25.8% in individuals 20–29 years), which may suggest a recent seroconversion. In addition, the interesting finding was a U-shaped association between age and seropositivity rate (higher seroprevalence in ≤29 and ≥60-year age groups compared to 30–59-year age groups), which is also seen in neuroborreliosis [29]. However, the cause of this observation is not explained.

The significant observation is a higher seroprevalence rate in patients with meningitis (44.7%) and febrile headaches (22.2%) compared with meningoencephalitis and myelitis (10.0% each), suggesting that BHAV disease is usually mild and the main presentation of the neuroinvasive disease is meningitis.

In Croatia, *H. punctata*, the main vector of BHAV, was recorded on ten different host species, mostly from ungulates, as well as one carnivore and one bird [14]. This species in Croatia was mainly recorded in localities in the Mediterranean biogeographic region, islands (Brač, Cres, Hvar, Krk, Lošinj), and in a littoral belt from Pula to Dubrovnik [3,7,30,31,32,33,34,35]. Recently, it was recorded at two localities in the Alpine biogeographic region (Gumance–Smrekova Draga, Kurjak-Udbina) [33,35] and six localities in the Continental biogeographic region (Donji Miholjac, Goričan, Koprivnica, Virovitica, Primišlje, and Požega) [36,37,38]. The first record of this species in the continental biogeographical region was made almost eighty years ago at localities of Gornja Posavina in an irregular quadrilateral Zagreb-Karlovac-Sisak (Petrinja)–Bjelovar [30].

*H. punctata* from Brač Island is the second tick species from which BHAV was isolated in 1977 [36]. Brač Island in central Dalmatia represents one of Europe’s eight natural foci of BHAV. Many shared biogeographic features were found among these BHAV natural foci: mean altitude is 200–850 m a.s.l.; frequent soil types are rendzines, terrae calcis, illimerized, and brown forest soils; agrarian type of landscape; and a Mediterranean climate with peak rainfall in the winter, dry, and hot summer. In central Europe, favorable habitats are xerothermic, which is microclimatically very close to the climate in southern Europe and its thermophilic vegetation type [3].

In the presented study, the majority of BHAV-seropositive patients resided at lower altitudes (less than 200 m a.s.l.). Still, in these regions, a significant increase in the prevalence with higher altitudes was observed. Vector-borne diseases are particularly sensitive to climate change since ectothermic arthropods have their internal temperature regulated by external environmental conditions. The extrinsic incubation period (replication of arboviruses within vectors) occurs faster at higher temperatures. Vector biting rates also tend to increase with temperature [39]. BHAV vector activity is likely limited due to lower temperatures at higher altitudes. The association of altitude and vector activity may not be so evident in the warmer Mediterranean climate, e.g., Brač Island, compared to the continental climate of inland Croatia. Some studies showed that prolonged extreme values of temperature (both high and low) adversely affect tick development by reducing their activity and increasing their mortality rate through desiccation [40], which could explain the negative association between BHAV prevalence rates and temperature as well as the number of warm and hot days observed in our study.

Additionally, BHAV prevalence rates positively correlated with population density. More time spent outdoors, whether for recreation (walking, running, excursions) or gathering various edible (asparagus) and medicinal plants (mint, mallow, elderberry), or fruits (blackberries, raspberries, chestnuts), and mushrooms, could be a contributing factor for higher seropositivity in counties with higher population densities. As a result, they are more frequently exposed to tick bites and tick-borne pathogens.

## 5. Conclusions

The detection of 20.8% of seropositive Croatian patients suggests that BHAV infections frequently remain unrecognized. Prevalence rates differed between counties. The majority of BHAV-seropositive patients were detected in patients who resided in areas at lower altitudes (less than 200 m). However, the prevalence rates increased significantly with higher altitudes in patients from these areas. In patients residing at lower altitudes, there was also a significant negative association between the prevalence rate and temperature and the number of warm/hot days. In contrast, precipitation amount, number of cold days, or cumulative insolation duration showed no influence on BHAV prevalence. Due to the small number of participants, further studies are needed to determine the prevalence and clinical significance of this neglected arbovirus in the Croatian population.

## Figures and Tables

**Table 1:** Demographic, epidemiological, and clinical characteristics of two patients with recent Bhanja bandavirus infection.

Characteristic		Patient 1	Patient 2
Demographic characteristics	Gender Age	Male 72 years	Male 60 years
Geographic characteristics	Area of residence Geographic coordinates	Rural, near the river N 46°17′10″; E 16°18′10″	Rural, near the river N 46°24′43″; E 16°37′30″
	UTM	33 T	33 T
	Altitude	177 m	146 m
Epidemiological characteristics	Risk factors	No data	Tick bite on the head during fishing two weeks before disease onset
	Date of disease onset	November 2018	June 2019
	Vaccination (tick-borne encephalitis, yellow fever)	No data	No
Clinical characteristics	Clinical presentation	Meningoencephalitis	Febrile headache
	Clinical symptoms	Fever, seizures, disorientation	Fever, headache, malaise
	Underlying diseases	Alcoholism	No
	Outcome	Improvement	Full recovery

UTM = Universal Transverse Mercator coordinate system.

**Table 2:** Laboratory and virology results of two patients with recent Bhanja bandavirus infection.

Parameter	Patient 1	Patient 2	Reference Range
Laboratory results (blood/CSF)			
L (Blood)	12.3	11.1	×10^9^/L
CRP (Blood)	22.8	22.6	<5 mg/L
Cells (CSF)	144	5	<5/mm^3^
Mononuclear cells (CSF)	85	–	100%
Proteins (CSF)	0.400	0.520	0.17–0.37 g/L
Glucose (CSF)	3.4	3.0	2.5–3.3 mmol/L
Virology results (serum/CSF/urine)			
RT-qPCR (CSF)	Negative	Negative	
RT-qPCR (Urine)	Negative	Negative	
VNT titer (Serum)	20	Negative	≥10 Positive
VNT titer (CSF)	20	20	≥5 Positive

L = leukocytes; CRP = C-reactive protein; CSF = cerebrospinal fluid; VNT = virus neutralization test.

**Table 3:** Prevalence of Bhanja bandavirus antibodies according to demographic and clinical characteristics.

Characteristic		Tested	BHAV NT Antibodies N (%)	95% CI	*p*
		N (%)			
Gender	Male	165 (64.9)	34 (20.6)	14.7–27.5	0.728
	Female	89 (35.1)	20 (22.4)	14.3–32.5	
Age group	<20 years	24 (9.4)	7 (29.1)	12.6–51.1	0.907
	20–29 years	31 (12.2)	8 (25.8)	11.8–44.6	
	30–39 years	43 (16.9)	9 (20.9)	10.0–36.0	
	40–49 years	23 (9.1)	5 (21.7)	7.4–43.7	
	50–59 years	33 (13.0)	5 (15.2)	3.4–28.2	
	60–69 years	49 (19.3)	10 (20.4)	10.2–34.3	
	≥70 years	51 (20.1)	10 (19.6)	9.8–33.1	
Area of residence	Urban	181 (71.2)	35 (19.3)	13.8–25.8	0.238
	Suburban/rural	73 (28.8)	19 (26.0)	16.4–37.6	
Clinical presentation	Febrile headache	18 (7.1)	4 (22.2)	6.4–47.6	<0.001
	Meningitis	76 (29.9)	34 (44.7)	33.3–56.5	
	Meningoencephalitis	150 (59.1)	15 (10.0)	5.7–15.9	
	Myelitis	10 (3.9)	1 (10.0)	2.5–44.5	

CI = confidence interval.

**Table 4:** Risk analysis for Bhanja bandavirus seropositivity.

Characteristic	OR	95% CI OR	*p*
Male (Ref.) vs. female gender	1.116	0.598–2.085	0.728
Age			
<20 years	Ref.		
20–29 years	0.844	0.256–2.783	0.781
30–39 years	0.642	0.204–2.023	0.450
40–49 years	0.674	0.179–2.538	0.560
50–59 years	0.433	0.118–1.585	0.206
60–69 years	0.622	0.193–1.814	0.407
≥70 years	0.592	0.774–1.814	0.359
Suburban/rural (Ref.) vs. urban area of residence	1.467	0.774–2.783	0.239
Clinical presentation			
Febrile headache	Ref.		
Meningitis	2.833	0.853–9.404	0.088
Meningoencephalitis	0.388	0.113–1.334	0.133
Myelitis	0.388	0.037–4.061	0.430

OR = odds ratio, CI = confidence interval.

**Table 5:** Climate and geographic data with Bhanja bandavirus prevalence rate in counties with tested patients.

County (Label)	Population Density (/km^2^)	Altitude (m)	Mean Temperature (°C)	N Cold Days (≤0 °C)	N Warm Days (≥25 °C)	N Hot Days (≥30 °C)	Insolation (h)	Total Precipitation (mm)	N Cases	Prevalence Rate
1	38.6	135	10.9	90	84	24	1948.9	809.3	0	0
2	64.0	92	12.2	93	93	31	1899.3	768.2	2	1.5
3	64.9	3	16.7	2	104	28	2636.5	1156.1	1	0.9
5	31.1	112	11.1	84	87	26	1908.8	1107.1	1	0.9
6	58.2	149	10.4	91	72	16	1974.1	795.6	3	2.9
7	98.8	203	10.4	92	70	16	2038.4	873	3	2.5
8	8.0	656	8.8	114	57	13	2018.2	1496.4	0	0
9	145.0	164	10.4	92	70	16	2038.4	873	6	5.7
10	62.5	94	11.1	82	93	32	1963.4	692.5	6	2.3
11	34.9	311	12.2	93	93	31	1899.3	768.2	0	0
12	74.4	13	14.2	18	87	27	2209.9	1554.2	1	0.4
13	31.4	98	11.2	82	84	24	1923.3	908.6	1	0.7
14	93.8	0	16.4	6	108	44	2637.3	785	0	0
16	127.1	173	10.4	92	70	16	2038.4	873	10	6.2
17	34.2	122	10.9	90	84	24	1948.9	809.3	0	0
18	59.0	108	11.1	82	93	32	1963.4	692.5	3	2.1
19	44.0	0	15.2	9	93	22	2578.4	911.8	0	0
20	97.9	158	11.6	59	73	17	1940	886	5	1.7
21	1200.6	158	11.6	59	73	17	1940	886	11	1.4

## Data Availability

Not applicable.
